# Low-Cost Road-Surface Classification System Based on Self-Organizing Maps

**DOI:** 10.3390/s20216009

**Published:** 2020-10-23

**Authors:** Ignacio Sánchez Andrades, Juan J. Castillo Aguilar, Juan M. Velasco García, Juan A. Cabrera Carrillo, Miguel Sánchez Lozano

**Affiliations:** 1 Department of Mechanical Engineering, University of Málaga, 29071 Málaga, Spain; nsanchez@uma.es (I.S.A.); juanmav@uma.es (J.M.V.G.); jcabrera@uma.es (J.A.C.C.); 2 Department of Mechanical Engineering, Miguel Hernández University of Elche, 03202 Elche, Spain; msanchez@umh.es

**Keywords:** data acquisition, vibrations, surface estimation, machine learning, automobile systems

## Abstract

Expanding the performance and autonomous-decision capability of driver-assistance systems is critical in today’s automotive engineering industry to help drivers and reduce accident incidence. It is essential to provide vehicles with the necessary perception systems, but without creating a prohibitively expensive product. In this area, the continuous and precise estimation of a road surface on which a vehicle moves is vital for many systems. This paper proposes a low-cost approach to solve this issue. The developed algorithm resorts to analysis of vibrations generated by the tyre-rolling movement to classify road surfaces, which allows for optimizing vehicular-safety-system performance. The signal is analyzed by means of machine-learning techniques, and the classification and estimation of the surface are carried out with the use of a self-organizing-map (SOM) algorithm. Real recordings of the vibration produced by tyre rolling on six different types of surface were used to generate the model. The efficiency of the proposed model (88.54%) and its speed of execution were compared with those of other classifiers in order to evaluate its performance.

## 1. Introduction

In the field of automobile research, many studies focus on estimating the type of surface on which vehicles move. The question is not trivial, since the improvement of the performance of cars, autonomous vehicles, or mobile robots depends on its correct determination. The automotive industry focuses a large part of its efforts on providing vehicles with the latest advances in perception or autonomous decision making in order to improve the performance of their systems and reduce or even the possible effects of an accident.

The precise determination of a road surface is fundamental for the improvement of vehicle dynamics. Many systems can be fed with this information, such as the antilock braking system (ABS), traction-control system (TCS), connected vehicles (V2V), and vehicle-to-road infrastructure (V2I). All require the most precise surface characteristics to be able to maximize their potential. Safety, comfort, performance, and traffic management are some of the areas that can be improved with the evolution of these technologies.

There are a multitude of approaches that attempt to solve this problem. Requirements demanded by the task sometimes collide with commercial interests, since they involve the use of systems or technologies that increase the final price of vehicles or are not developed enough to be considered useful.

This work proposes a system composed of a single low-cost accelerometer that is used to measure the vibrations produced by tyres when they roll into contact with different surfaces. The vibration approach is presented as a viable, simple, and economical solution. Excitation caused by the surface of a tyre is analyzed to discriminate six different types of surface by means of a classification algorithm. Information is processed in time and frequency domains for the extraction of characteristics that allow for the successful evaluation and classification of the surface. The classification task is carried out by means of a self-organizing-map (SOM) algorithm [1,2,3] that allows for the processing of large information datasets and their dimensional reduction to a 2D visual space.

The SOM algorithm allows for the dimensional reduction of problems with multiple input factors. This allows for the information to be visually analyzed, which improves its analysis and interpretation. The SOM algorithm is capable of generating clusters of data that have similarities generating topological relationships on a predefined grid using the Unsupervised Learning paradigm [4,5]. The topology of the generated network makes it possible to discern whether or not a surface belongs to each of the generated clusters. For this purpose, the proposed validation criterion was based on SOM operating principles and, Voronoi tessellation [6] was used for the task of separating the different created groups. Lastly, the obtained results by the proposed algorithm were compared with those of similar algorithms to evaluate its effectiveness.

The contributions of this work with respect to existing approaches are:

- development of a continuous road-surface-estimation algorithm based on the use of self-organized maps (SOM);
- use of the vibration produced in the suspension system of a real test vehicle by the rolling of the tyre for surface-classification purposes;
- development of the signal-processing method and selection of most favorable parameters to achieve the best classification performance;
- comparison of the proposed methodology with other common machine-learning algorithms (classification ratios and execution times were obtained to evaluate the performance of the proposed model).

The remainder of this article is organized as follows. In Section 2, a review of the related works is included. Section 3 describes the framework devoted to the acquisition and processing of information. Section 4 explains in detail the chosen feature-learning methodology and implementation approach. Section 5 shows the performed experiments and simulations with the generated classification models, and their evaluation with performance tests. Section 6 shows the assessment of the obtained results and a discussion of the implementation modes. Lastly, in Section 7, conclusions and proposals for future work are drawn.

## 2. Related Research

This section presents a review of the related literature focused on developed approaches and methodologies to solve the surface-estimation problem described in the introduction. The used methodologies are varied, but it is well known that they are classified into two large groups depending on the approach used for the problem.

We can find technologies of an exteroceptive nature that focus on studying which factors affect the state of the surface and its characteristics (cause-based methods). This includes technologies based on the use of optical sensors, cameras, infrared cameras, lasers, and ultrasonic sensors. This type of method has the advantage of being able to provide surface information even when the vehicle is stationary. In [7], a real-time processing system for surface classification using images captured by CCD cameras is proposed. The authors used a wide dataset covering different lighting ranges or weather conditions. This denotes one of the main problems of the robustness of this kind of systems, namely, the dependence on lighting conditions and the need for large information datasets.

This segment experienced an important advancement with the development and inclusion of artificial-intelligence (AI) algorithms to the process. The introduction of convolutional neural networks (CNNs) has allowed for the development of more accurate models in image analysis thanks to their capacity to extract and analyze characteristics and patterns [8]. In [9,10], the authors explored the possibility of taking advantage of surface-image recognition to estimate the adhesion coefficient using tyre models. Sensor fusion is also used as a strategy to monitor surface conditions with the use of image analysis and ultrasonic sensors [11]. These systems have the clear disadvantage of using expensive sensors that are not included in a standard sensor set of a conventional car.

There are also technologies of a proprioceptive nature (effects-based methods). These technologies analyze which effects are produced when the vehicle moves on different surfaces, and thus carry out their classification. This branch can be divided into approaches of acoustic analysis, vibrations, sliding, study of deformations, and other, more typical of mobile robotics approaches such as haptic systems. In [12,13], the sound generated by articulated, tracked, and wheeled robots in their interaction with the environment was studied. The authors proposed a method in which recorded noise is processed to prepare the signal prior to its classification by a support vector machine (SVM). Within vehicle dynamics, works such as that of Masino et al. [14] studied sound from inside the tyre cavity with which, thanks to the use of a multilayer perceptron (MLP), it is able to classify surface and climatic conditions. The acoustic approach has a weak point that hinders its use: the presence of numerous and varied external acoustic interferences to the noise of interest.

Due to noise-approach drawbacks, vibration analysis is presented as an alternative in the current literature. This procedure achieves a better response than that of acoustic systems since it is affected by more easily controllable phenomena, such as gravity and the rigidity or damping of the contact elements between vehicle and surface. In mobile robotics, the autonomous skills of robots to recognize surfaces and classify them appropriately is addressed in [15,16]. In the automotive field, we are working on the reconstruction of a surface profile by measuring an accelerometer and the position of the vehicle integrated by global positioning system (GPS) [17,18]. Analysis in the time spectrum of the surface profile and its subsequent classification by means of an SVM completes the proposed classification algorithm.

Creating datasets that cover the entire spectrum of surfaces, climatic conditions, or speeds involves a great deal of acquisition and processing work. In [19], it was proposed to interpolate surface information on the basis of particular events. It was demonstrated that, although the solution is not as effective as the real recording of a surface, the proposed solution helps to have a wider and more dynamic system without the need to make new measurements.

The use of neural networks and fuzzy logic in surface-classification tasks is also presented. Dupont et al. [20] used a probabilistic neural network (PNN) as a classification tool. In [21], the same network model was used in combination with an unscented Kalman filter (UKF) to improve the robustness of classification and vehicle-state estimation. Another network variant known as radial basis function (RBF), commonly used in model fitting, series prediction, and classification problems, was used as a baseline of a classifier in [22]. Apart from the one in [23], surface recognition is used to adapt a semiactive suspension system through the use of models that use a fuzzy kernel.

In [24,25,26], the most common machine-learning classifiers used in surface-classification tasks were analyzed. Moreover, works such as Park et al. [27] or Mei et al. [28] addressed the capabilities offered by deep learning in pattern analysis and feature extraction. The use of long short-term memory (LSTM) networks in combination with CNNs or sensor-fusion techniques reinforce the knowledge of the trained models.

The described methodology presents a quality that offers a competitive advantage compared to classical procedures of surface classification based on the study of the slip, as it is shown in [29]. In [30], tyre slip was also used as a performance characteristic. As a result of the combined use of fuzzy logic and neural networks, the estimation of the surface-adhesion coefficient and its classification were achieved. This kind of approach requires the tyre to slip, and this involves, in many cases, risky situations (acceleration or braking). In addition, the reaction of the system occurs after the slip is initiated, so the response of the system may be late. The described procedures provide information without requiring tyre slippage, and anticipate a potentially dangerous situation.

Focusing attention on the proposed methodology for this work, self-organised maps are presented as a versatile and simple algorithm that allows for the manipulation of a great amount of information, offering easy visualization of the results. These facts, among others, make it a viable and widely used methodology in disciplines such as image analysis, voice recognition, process control, artificial intelligence, and data mining.

The SOM algorithm is utilized in classification tasks, as shown by Xia et al. [31] with their work on the classification and grouping of lithium cells in a production line. The different variants and learning paradigms of this algorithm are also applicable in satellite-image classification [32,33] and its use in image segmentation for the improvement of object detection in video surveillance [34].

Lastly, there are also examples of the use of this type of algorithm within the automotive industry. Gil et al. [35] used an SOM to explore the possible design relationships on the basis of objective factors and the subjective sensations of the user. This point of view allows for the design of vehicles to evolve on the basis of user experience. In addition, this algorithm is used in areas such as the maintenance of road infrastructure for use in surface assessment and the detection of possible cracks [36].

As was mentioned before, the proposed algorithm in this paper uses an SOM to perform surface classification using the vibration signal measured in the vehicle-suspension system. This approach provides a simple, fast, and reliable solution to this problem.

## 3. Parameter Acquisition and Data Processing

**Figure 1:**
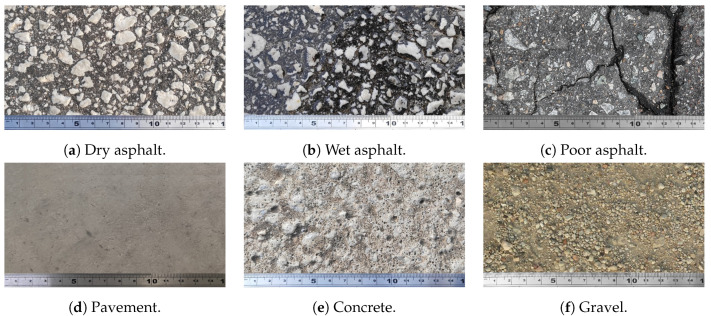
Sample of test surfaces.

**Figure 2:**
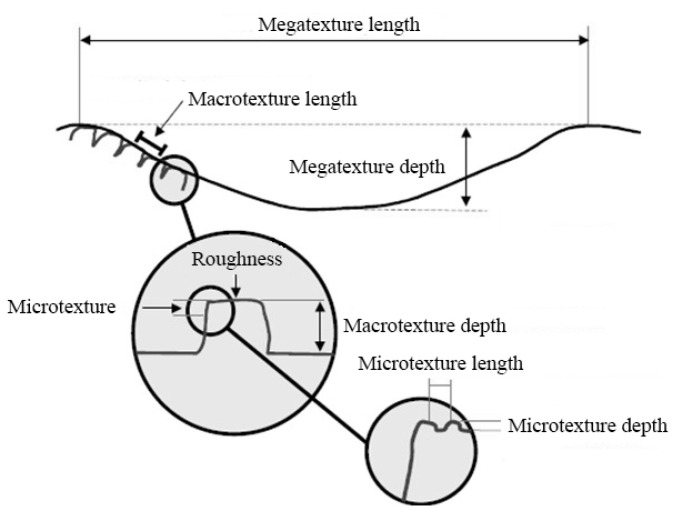
Representation of different textures defined for a surface (adapted from Piarc, 1995; Highways Agency, 1999a).

In this section, the process of obtaining datasets is analyzed and described. The extraction of the necessary surface parameters to efficiently execute the classification algorithm developed in this work is addressed. A set of surfaces (see Figure 1) with different characteristics were tested in this study in order to develop a classification model capable of identifying at all times the surface on which a vehicle is located. Table 1 shows a summary of each analyzed surface:

The vibrations produced by each surface were recorded during the tests, obtaining a time signal with the characteristics of each surface. Tests were based on recording the vibrations produced at the base of the vehicle suspension by the tyre rolling onto the surface. Analysis was focused on the excitation generated on the vehicle due to the macrotexture characteristics of each surface. The microtexture was excluded from this study because it has a range that is unapproachable for this type of system. On the other hand, the megatexture could not be considered as a representative feature of a surface either, but as a particular place or situation of a surface. Table 2 shows the defined dimensional range for the surface texture according to international standard ISO 13473-1 [37]:

Figure 2 shows that the macrotexture is located in the middle range of the commonly established surface textures. Working in this range allows for the characterization and identification of each surface with the available methods. This texture is formed by the grain dimension that makes up the surface. The geometry of the grain, its separation, and the compaction of the elements are predominant. On the other hand, the conditions in which the analyzed surface is found have an important influence level. The presence of water, snow, loose gravel, or fissures can significantly influence the vibration produced in the tyre.

From the study of these signals, and the extraction of outstanding and differential characteristics, the working datasets for the generated models were elaborated. The measurement system and methodology proposed in this document are described in the following subsections.

### 3.1. Data-Acquisition System

**Figure 3:**
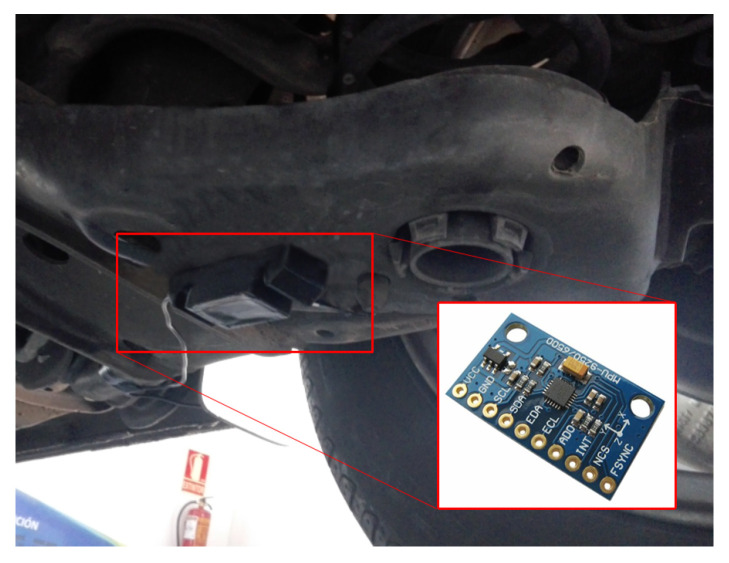
Location of the measuring system.

Data acquisition in a real vehicle was carried out in this work for the development of the proposed system. General-purpose elements were used with the premise of developing an efficient and economic system. The system consisted of an accelerometer for taking measurements, an acquisition system (based on a microcontroller), and a computer for storing the tests. The implementation was carried out as described below:

1. Accelerometer
  An MPU-9250 sensor was used as an acquisition element of the variables of interest. It was a 9 degree-of-freedom (9DOF) inertial measurement unit manufactured by InveSense Inc. (TDK Corp., San José, CA, USA) based on a microelectromechanical-system (MEMS) design. An internal configuration was established that maintained a refresh rate of the measured variable equal to or greater than 1 kHz.
  This sensor had an analog-to-digital converter (ADC) with an adjustable range (see Table 3). The range was established in the ±4 g option (8192 LSB/g), suitable for estimated operation range.
2. Microcontroller
  The electronic bench chosen for this work was an Esp32 microcontroller (two 32-bit cores). It is a system on chip (SoC) designed by Espressif Systems Co., Ltd. (Shanghai, China). The module communicates with the accelerometer and sends the information to the computer.
  The development of the code was based on the use of interrupt service routines (ISR) to ensure system runtime. An acquisition frequency of 1 kHz was set for signal processing.
3. PC host
  The measurement system was connected to a PC that acted as a storage base for the tests. Communication with the main system was established through a digital bus (UART). The working software chosen for this task was LabVIEW^TM^ 2018 (National Instruments, Austin, TX, USA) thanks to which the data were shown and processed in graphic form during the tests.
4. Test vehicle and measurement system location:
  The accelerometer was firmly attached to the surface of the rear-suspension arm. Figure 3 shows the location of the sensor on the test vehicle.
  The deviation from the vertical reference observed during the tests averages $2.4^{\circ }$, when the vehicle is under standard load conditions, with two occupants and a full tank. For this reason, the effect produced by this deviation can be considered negligible compared to other factors that may influence the measurement.

### 3.2. Variable of Interest

By carrying out tests on a real vehicle, information was collected from the different types of surfaces with the accelerometer mounted on its rear-suspension arm. Only vertical acceleration $\left(a_{z}\right)$ was used as a working variable in this work.

The effects produced by the presence of the tyre and the vehicle’s suspension could not be ignored. To this end, certain parameters were kept invariable during the tests in order to control their influence on the collected information. In this way, the speed in which the tests are carried out was set at 50 km/h on all studied surfaces. Second, vehicle load was kept at two occupants. Third, tire pressure was kept constant during all tests to mitigate or control the influence of tyre damping on the recordings. To this end, pressure of 2.2 bar was set on all tyres for the duration of the working session. Lastly, tests were carried out on straight sections of the road.

Given the established test conditions and the 205/55 R16V tyre, with an acquisition frequency of 1 kHz, appreciation of the macrotexture conditions could be covered. The tyre performed 7 revolutions per second at the established speed for the tests. Therefore, measurements could be obtained at approximately every 2.5° thanks to the acquisition frequency of 1 kHz. The size relationship between texture and tyre was the most appropriate to be measured given the elasticity of the tyre and the grain size of the surface.

**Figure 4:**
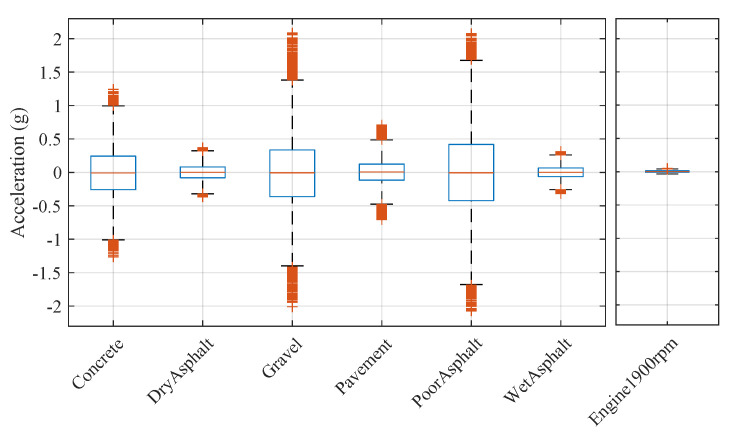
Signal-magnitude comparison for each surface. Case is represented in which the vehicle was at a standstill and the engine was revving at 1900 rpm (Engine1900 rpm).

Differences in the distribution of the acquired signals on the different surfaces could be observed in Figure 4. Additionally, signal magnitude recorded in the test in which the vehicle was at a standstill and engine speed was raised to a steady 1900 rpm (similar to the engine speed during the dynamic tests) is also represented on the right side of the figure. The magnitude of the vibration measured in this case was of a lower order than the one obtained in the conducted tests on the test surfaces, so its influence could be considered negligible.

**Figure 5:**
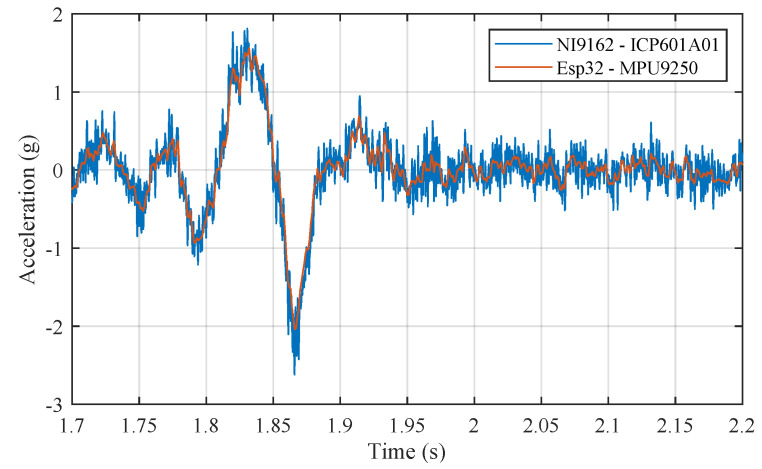
Comparative test for validation of accelerometer measurements.

Lastly, as Figure 5 shows, a comparative test was carried out with a high-quality sensor to check the fidelity of the acquired signal. A calibrated ICP 601A01 accelerometer from manufacturer IMI Sensors (PCB Piezotronics Inc., Depew, NY, USA) and an NI-9233 acquisition card from National Instruments were used for this purpose. The signals from the two accelerometers provided similar results. It was demonstrated that the low-cost accelerometer could capture the vibration signal as the reference accelerometer, but with a lower appreciation given its acquisition frequency. The observed dispersion in the signal of the ICP 601A01 was due to its higher sampling frequency (50 kHz).

### 3.3. Data Processing

The processing of the tests began with cleaning the signal and its segmentation. Then, extraction of the characteristics for each extracted segment was performed, which would later be used by the system for the classification task.

#### 3.3.1. Processing and Segmentation

The acquired signal by the accelerometer was affected by gravity, so this component had to be eliminated. For this purpose, the mean value in a static case with the same sensor orientation was subtracted from the measured data. Additionally, the signal was filtered to remove spurious elements that differed from the normal distribution of the signal. To do so, all measured data whose values were outside an interval defined by three standard deviations from the mean were removed.

At a later stage of this preprocessing, the signal was analyzed in segments or windows (w). The influence of window size on the final classification results has to be considered, and the frequency with which the prediction made by the system would be updated. During the training phase, the estimation system could be implemented with overlap (%) between observation windows. This allowed for a greater variety of observation windows, which improved the generalization capacity and robustness of the model.

#### 3.3.2. Feature Extraction and Selection

The application of the learning algorithms required the analysis and extraction of characteristics from the working signals. The classification system had to be fed with a set of representative data of each surface (see Table 4); so, in this case, the direct signal was not enough.

The dataset supplied to the system was composed of a vector of characteristics associated with each input window. Analysis developed in this work contemplated the use of characteristics in the time and frequency domains of the signal. Of all the studied parameters, only a selection of the most representative ones composed the input vector of the system in the training phase.

As shown in the table above, $p_{t}$ are the features of the processed data in the time domain applied to each segmentation window, $p_{fa}$ represents the characteristics extracted from the frequency domain considering the amplitude spectrum, and $p_{fp}$ corresponds to features of the power spectrum.

Once each of the evaluated characteristics was analyzed, separate normalization was carried out for each between [−1,1]. The different scales and units of each selected parameter may have had an adverse effect on the model fit. This normalization reduced the aforementioned effect and made the provided information of each variable as representative as possible.

## 4. Learning Process—Self-Organizing Maps (SOM)

The elaborated classification procedure in this paper addresses the task of classifying surfaces from acceleration-sensor data using classification tools based on artificial neural networks (ANNs). The purpose of this classifier is to provide vehicle systems with the ability to perceive the road surface on which the vehicle is continuously moving.

Surface recognition is implemented in two phases. First, an unsupervised-learning process is carried out using a self-organizing map (SOM) as a tool. This model explored the characteristics of the signal to produce a cluster map of all provided information. In the second phase, the recognition and labelling processes of each region are developed. This segmentation of the map based on Voronoi tessellation [6] was used to determine whether new information belonged to one section or another. After the second phase, the algorithm could perform the surface-classification task in real time from the measured data.

**Figure 6:**
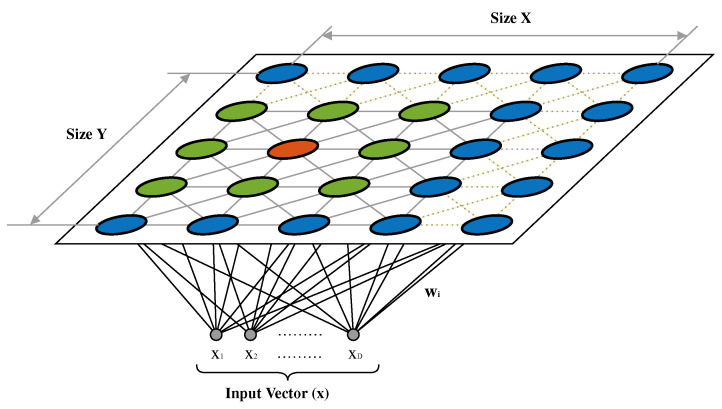
Self-organizing-map structure.

Self-organizing maps are a well-known clustering algorithm developed by Teuvo Kohonen [1]. Its application is common in fields such as economics, geography, engineering and, in short, data analysis in time series or categorical data. Another notable feature of this type of algorithm is the reduction of the input data to a low-dimensional space, generally two dimensions. As shown in Figure 6, the discretized representation of the output space is known as a map.

This kind of algorithm presents two major differences compared to other neural-based techniques. First, the map is composed of interconnected nodes (neurons) that form a grid. This internal structure makes them different from the commonly used layer structure in other algorithms such as Multilayer Perceptron (MLP). Second, self-organizing maps employ competitive learning as a learning technique, as opposed to error correction based on methods such as descending gradient. It is a simple implementation algorithm whose complexity is linear with respect to the volume of work data, which allows its use in problems where there is a large amount of data and complexity.

The initial map presents random weight distribution, so it is recommended to randomly consign the seed in those processes that require repeatability. The final intention of this mode of operation is to make the obtained results independent from the initial weight distribution.

Summary of contributions from the use of this method:

- Dimensional reduction and clustering. Distribution on a two-dimensional map favors the visual analysis of complex data.
- Unsupervised learning allows for working with data for which not all characteristics or properties are initially known.
- Reduced complexity of the algorithm.
- Quantification property. Models represent the data space as accurately as possible.
- Self-organizing property. Models retain the topology of the input data.

### 4.1. Learning Stages

The SOM algorithm is composed of two main stages, the competitive and cooperative stages. In the first, the neuron closest to the input value is located, and in the second, the weight of that neuron and that of its closest neighbors is updated.

#### 4.1.1. Competitive Stage

The number of neurons needed in the input layer is determined by the dimensionality of the problem to be analyzed, according to the number of parameters that compose input vector x(t), where *D* is the dimension of the vector:

(1) $$x\left(t\right)=\left[p_{1},p_{2},\ldots ,p_{D}\right]$$

The output layer (map) is composed of an arbitrarily sized two-dimensional matrix of neurons defined by the design criteria. Both neural layers are fully connected, so that each input value can be reflected in each of the output neurons. The match is total and affects the neighboring region.

The used notation throughout this article is as follows. Total number of neurons in the output layer is *N*, where N=a·b, a and b being the number of nodes along the *X* and *Y* dimensions of the SOM structure, respectively. The distance between any two neurons *i* and *j* is defined by Euclidean distance with two $r_{i},r_{j}\in R^{2}$ positions in the generated lattice:

(2) $$d\left(i,j\right)=∥r_{i}-r_{j}∥$$

where x(t) is each of the entries to the system, and ω(t) is the representation of weight vectors associated to neurons of the network. At each step of time (t), a new sample is introduced into the network, and a winning neuron is declared. The winning neuron is determined by the minimal found distance,. and its closest neurons suffer the readjustment of their weight vector; $\omega_{i}\left(t\right)$∈$R^{D}$, where *D* is the dimension of the input space.

In this stage, several phases of execution are distinguished:

- Map initialization: the weight vector of the output neurons is automatically generated with randomly normalized values. As previously mentioned, in the case of interest in a deterministic output, the random generator has to be set. This allows for having an identical distribution pattern each time and thus being able to correctly evaluate the influence of the input data on the final classification result.
- Normalization of input data.
- Location of the winning neuron (best-matching unit (BMU)):
  (3) $$r_{BMU}\left(x\left(t\right)\right)=\arg\min_{i\in \{1,\ldots ,N\}}∥x\left(t\right)-\omega_{i}\left(t\right)∥$$

#### 4.1.2. Cooperative Stage

In this stage, the topographical distribution of the neurons is modified according to coincidence with the inputs, and the update of their weight and their neighboring nodes. During the learning process, the weight of the vector of the winning neuron is updated. Next, the weights of their closest neighbors are updated with the neighborhood function, which represents the reaction of neighboring neurons to similar input values. The neighborhood function tries to preserve the topological distribution of the input data.

Execution stages:

- Definition of the region of influence: neighbors affected by the zone of maximal coincidence with the winning neuron are determined by establishing a neighborhood radius σ(t), for each execution cycle. The following expression defines the weights of the distances of the evaluated neighbors in the generated grid (2D):
  (4) $$h_{BMU,i}\left(t\right)=\exp\left(-\frac{d{\left(r_{BMU},r_{i}\right)}^{2}}{2\cdot \sigma {\left(t\right)}^{2}}\right)$$
- Adjustment of weights of neighboring neurons:
  (5) $$\omega_{i}\left(t+1\right)=\omega_{i}\left(t\right)+\alpha \left(t\right)\cdot h_{BMU,i}\left(t\right)\cdot \left(x\left(t\right)-\omega_{i}\left(t\right)\right),$$
  where α(t) is a decaying learning rate at the neighborhood.
  (6) α(t+1)≤α(t)

Usually, learning algorithms are based on updating or adjusting weights on the basis of the descending gradient of an energy function. However, the SOM algorithm does not implement the descending gradient as the basis of operation. It employs mean squared error (MSE) due to the quantization of the inputs within the Voronoi regions generated around each node:

(7) $$\mathrm{MSE}=\frac{1}{K}\sum_{k=1}^{K}\min_{i\in \{1,\ldots ,N\}}{∥x_{k}-\omega_{i}∥}^{2},$$

where *K* is the number of input samples.

### 4.2. Learning Paradigm—Unsupervised Learning (Clustering)

**Figure 7:**
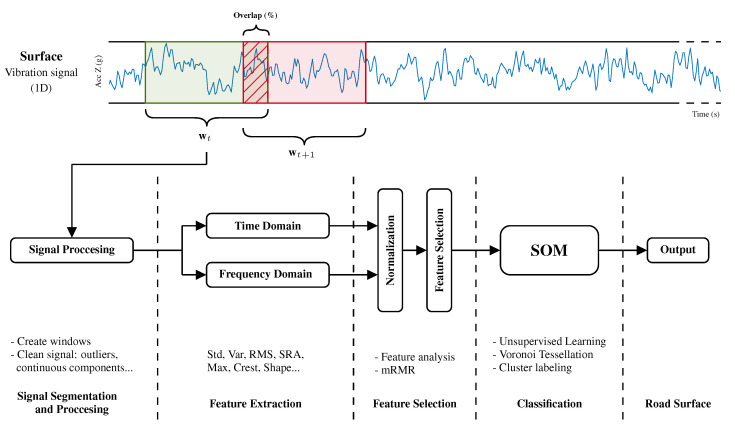
Workflow diagram of proposed classifier based on self-organizing maps.

In the first instance, the SOM algorithm is fed with an unlabeled surface data (see Figure 7), and the extracted features for each surface are grouped into input vector x(t).

This stage has an exploratory character. Only input data are known without having a correspondence with output data (unsupervised learning). Therefore, the system can only describe the structure of the data or find some kind of organization (clusters). The system explores similarities in the data to form clusters, but it does not guarantee that these are accurate or meaningful. This allows for new correlations that were not expected to be explored in the data.

The map resulting from training is used as a basis for classification and, in a second stage, the system is provided with the labelling of the generated regions. On the basis of the same principle as that of the SOM, the Voronoi sections are established as a criterion for the segmentation of the obtained map.

Once the map is trained and the Voronoi sections are generated, each section is identified and labelled. This step produces a complete system capable of classifying an unknown entry by assigning it to one of the previously created sections.

## 5. Experiments and Results

With the vibration data recorded during the tests on the different surfaces, the evaluation and validation of the proposed classification model, and analysis of the obtained results are carried out. The signal is continuously processed with a frequency of 1 kHz, while the classification system works on predetermined time windows.

The changing nature of an SOM requires precision during the initial stage of training. Once the SOM is defined in a deterministic way, the evolution of the data and the learning process itself define the final outcome. Despite possible changes in the map topology, data with similar characteristics end up being grouped in nearby regions. This factor is vital to achieve an equal clustering map in front of the same input data and thus be able to evaluate the influence of other factors.

### 5.1. Training Phase

To guarantee an equitable representation of each surface on the generated map topology, the training data series for each case was checked. On this basis, a dataset composed of the different time series of approximately 8 s for each of the 6 studied surfaces is available.

Taking the largest possible window size (0.3 s) and considering a 35% overlap, a total of 39 windows (buffers) were achieved for each surface.

The data series was also divided into two batches to rigorously evaluate the classification capacity of the system with data not used during this phase. For training, 60% of the data were used, while the remaining 40% were used for the validation test. This separation favors the final performance of the system and helps to avoid the appearance of overfitting on the model. Similarly, a random disorder was generated in the different buffers of each batch to favor the system’s generalization capacity. The model did not intensively specialize in training data.

Another factor to consider is the size of the used map. Dimensioning the map for the classification task has to evolve as the training process advances because of the lack of dimensioning criteria for the SOM map. There are some rules to approximate an adequate map size:

- Avoid large maps where the volume of processed data leaves a high percentage of nodes empty.
- Increase the size of the map enough to have good representation of the input data, avoiding excessive agglomerations in the same group of nodes.

For this work, six different surfaces were studied and are represented by 10 characteristic parameters. This resulted in an input matrix with dimensions of 10 × 39 for each one.

A grid size of 13 × 13 nodes was estimated for the map considering the criteria mentioned above. An occupation percentage of more than 70% and an appropriate separation of the areas of interest were achieved with this size. In Section 5.3, the dimensionality of all problem parameters is studied.

The execution of the model is done on MATLAB (R2019b, Natick, MA, USA). It is completed with hexagonal topology distribution and an initial neighborhood distance (σ) of 6. The learning factor (α) of the competitive network was initialized to 0.01 (by default).

#### Validation Criterion

**Figure 8:**
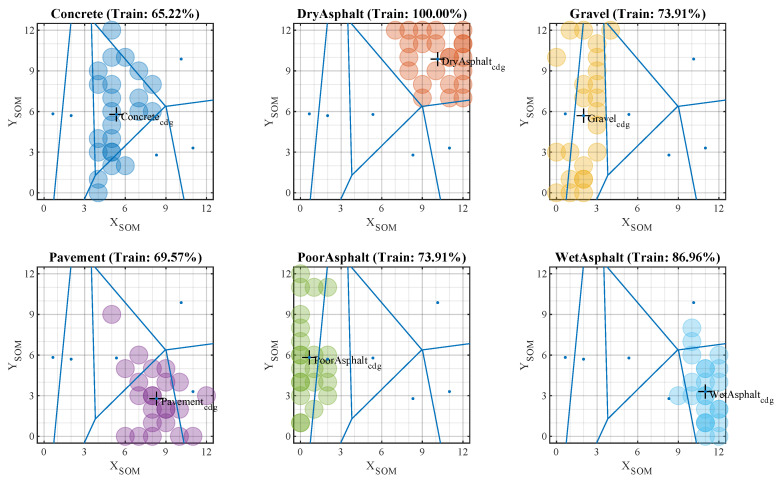
Sample classification using self-organizing maps—training phase (cluster generation).

Once the training phase is completed, the map shows the different clusters associated with each surface. The next step is to establish a validation criterion that allows for the classification of new inputs to the model. As shown in Figure 8, the chosen criterion for this purpose was Voronoi tessellation.

At this point, the work was concentrated on determining whether the node associated with each new vector belonged to one of the clusters (Z) formed during the training phase and with its associated label:

(8) $$j\in \left\{1,2,\cdots ,Z\right\}/\min_{j}∥r_{i}-r_{cdg_{j}}∥$$

This method generates a series of separation regions by tracing bisectors of the segment that joined the centers of gravity of the different formed clusters. The two-dimensional polygons generated around each reference point were equidistant from their immediate neighbors. Making use of this property, any point in a region could be associated with the label assigned to its center of gravity. The location of each SOM input is represented by a colored dot (see Figure 8). Similarly, different colors represent different surfaces.

### 5.2. Test Phase

**Figure 9:**
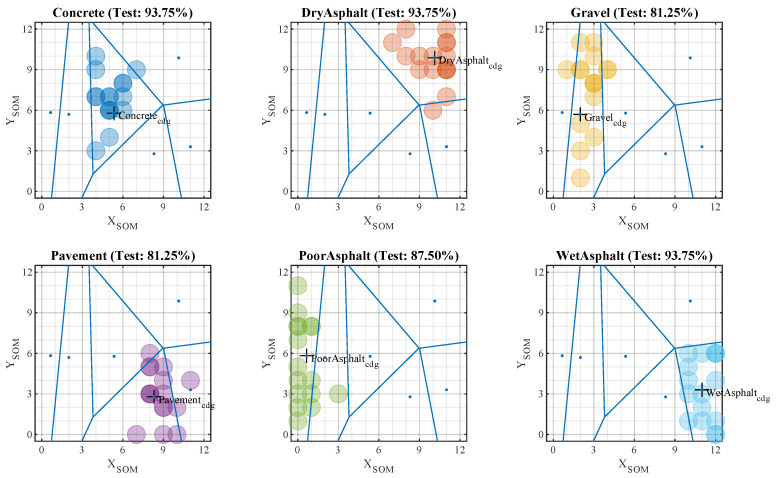
Sample classification using self-organizing maps—test phase (validation data).

To evaluate the capacity of the classifier, we used the initially separated test data. These data were not used in the previous learning phase and were labeled. With each input vector, a label was obtained as a result of the classification, and this could be checked with the actual label. Figure 9 shows that the centers of gravity of the training clusters and the generated boundaries were preserved at this stage.

**Figure 10:**
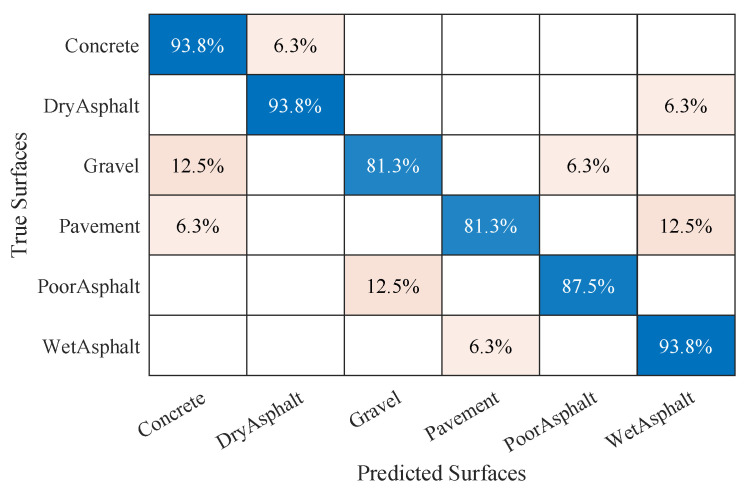
Confusion chart corresponding to estimation rate achieved in six-surface experiment.

To analyze the overall results of the test, a confusion matrix was generated. Hence, the success rates achieved by the model for each surface could be checked. The final results per surface are shown in Figure 10. The confusion matrix resulting from the test phase shows how the proposed model achieved adequate classification of all surfaces, with an average result of 88.54% over the test data.

### 5.3. Model Performance

In this section, the classification capacity of the SOM is evaluated employing a direct comparison with multiple classifiers. The random seed chosen for the initial distribution of weights in the SOM was fundamental to achieve a good classification. It is advisable to perform multiple tests with different seeds before adjusting the setting parameters. After the conclusion of the training with different random seeds, the SOM ended with the grouping of the different datasets, but there could be seeds that generate a better or worse final result.

Table 5 summarizes the range of use of the available parameters for the optimization of the proposed classifier:

In machine-learning algorithms, results commonly show high dependence on the working datasets that decreases as the volume of the dataset increases. A 35% overlap in the observation windows is used in order to reduce this dependence. In addition, the random permutation between different windows is separately generated for each batch to ensure better generalization.

The number of windows is limited to achieve comparable results between different methods and observation times. For the most unfavorable case considered in this work (0.3 s), a total of 39 buffers were created for each surface. Of the training data, 60% were previously established for each surface, so 23 buffers were for the generation of the model, and 16 buffers were for its validation in the final phase of the test.

**Figure 11:**
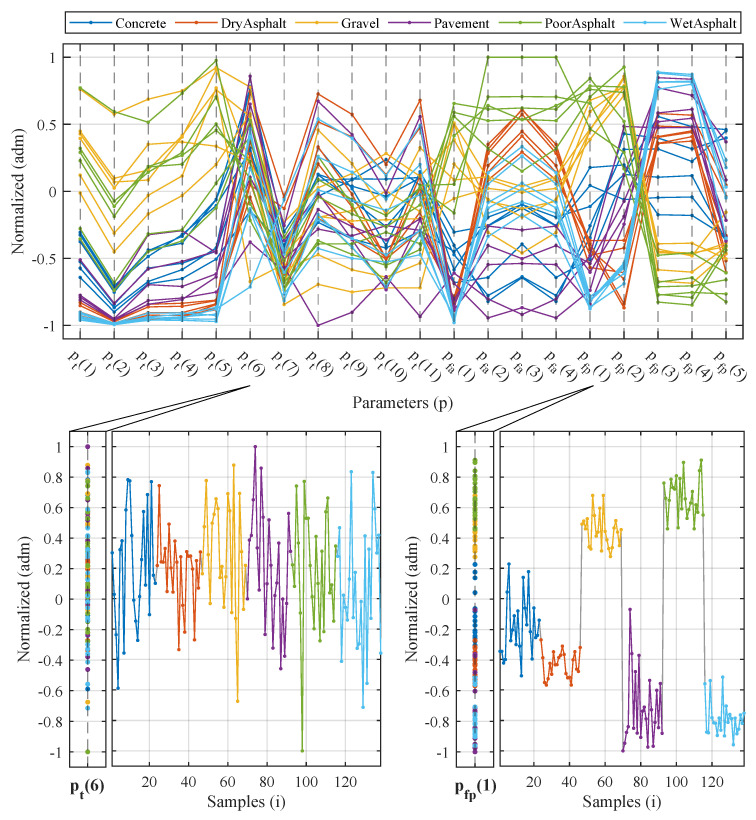
Parameter inspection sample with (**right**) good and (**left**) bad rating responses.

Another factor to consider is the quantity and quality of the inputs (see Figure 11). After analysis and evaluation of all initially proposed inputs, those variables that are more decisive for the task can be selected.

Those characteristics that better separate different surfaces were selected. Recommendations of the minimum-redundancy maximum-relevance (mRMR) were also considered for the selection of the most representative characteristics. An input vector composed of 10 elements was generated as a result of this analysis:

(9) $$x\left(t\right)=[p_{t}\left(1\right),p_{t}\left(2\right),p_{t}\left(3\right),p_{t}\left(4\right),p_{t}\left(5\right),p_{fa}\left(1\right),p_{fp}\left(1\right),p_{fp}\left(2\right),p_{fp}\left(3\right),p_{fp}\left(4\right)],$$

where parameters $p_{t}$, $p_{fa}$ and $p_{fp}$ were previously described in Table 4.

**Figure 12:**
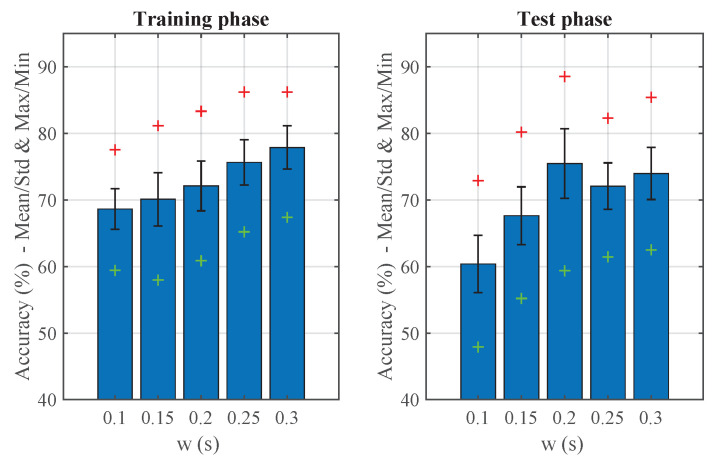
Summary of self-organizing-map (SOM) performance search results.

After completing the search for optimal SOM configuration parameters, analysis of the results revealed a solution that achieved 88.54% performance in the test phase. Consider the dispersion of results as a range that includes both good and bad configuration cases. A summary of the case study can be seen in Figure 12, and below summarizes the results obtained for a 13 × 13 element map. The optimal value was achieved with an observation window time setting of 0.2 s, and for a learning parameter setting of 6 initial neighbors and 250 iterations per epoch.

As window time was increased, the algorithm improved its performance during the training phase, but the test results declined. This can be because the seed chosen for training was suitable for the presented configuration or the appearance of an overfitting phenomenon. In this case, the model specializes in training data but, as can be seen, prediction results on the test data were worse.

As an evaluation of the proposed method, the generated map was tested in direct comparison with other classifiers. Table 6 shows the comparison of the obtained classification ratios. In this table, different types of classifiers with generic configurations were included, all of them available in MATLAB’s Classification Learner. The comparison was carried out over the same dataset with which SOM training and validation was carried out. All other parameters influencing the test remained unchanged. A total of 100 iterations were performed to obtain representative statistics of the performance of the different algorithms. The table shows the results for the training phase and the validation results of the different models with the test data. Lastly, the execution times required for each model for the classification of the complete dataset and its comparative ratio to the proposed model are shown.

The experiment for analyzing the classification ratios and execution times was carried out on an Intel(R) Core i7-9700K CPU, 3.6 GHz, 32 GB RAM machine with an NVidia GeForce GTX 1660 Super GPU with 6 GB of VRAM.

## 6. Discussion

An intensive search was made to optimize the dimensional and learning parameters of the SOM to achieve an appropriate level of classification.

As a previous step of the training phase, the acquired information was processed in an effort to make the best use of the available data. First, outliers that were far from the common dispersion for each surface were cleaned. Second, data were normalized to ensure the same impact on the model. Lastly, information was divided into training and test data with the appropriate duration to achieve the desired system performance (60%–40%). The parameters that made up the input data matrix were analyzed to reduce data that were not representative or were redundant.

One of the main future objectives is to incorporate the model into the control systems of a vehicle, so its agility has to be sufficient to ensure that the task of determining the surface does not become a bottleneck. Comparatively, as Table 6 shows, with optimization carried out, a competitive solution to the proposed classification problem was achieved (88.54%, 3.20 ms). Only the quadratic discriminant classifiers (92.71%, Exe. Ratio 3.19) and Gaussian naïve Bayes (89.58%, exe. ratio 2.55) achieved a balance between classification and the comparable execution time. The efficiency gap of these values was not more than 5% with the best obtained results. The rest of the classifiers that presented execution times of a higher order than that of the proposed model could be discarded. This can be a limiting factor when carrying out real-time control tasks on vehicle systems. However, execution times depend on the system where it is executed and its optimization.

Another aspect to consider is the time of observation. Increasing window time may lead one to believe that the effectiveness of the model increases, but it may also generate results with a high dependence on past information. The current estimate should be as representative as possible of the current situation of the vehicle. A balance has to be found between observation time and model execution time.

Making use of the two-dimensional representation of the SOM, visual analysis of the clustering of different surfaces can be carried out. After training, each section is identified, and the map is converted into a complete classification system.

The dispersion of each studied surface could be analyzed on the same map (see Figure 8 and Figure 9). This made it possible to see that more compact or better-formed surfaces were well-delimited by the model and grouped in more closed clusters in such cases as pavement or asphalt. On the other hand, surfaces such as gravel or poor asphalt have more irregular and varied surfaces, so the map groups them into more dispersed clusters.

Lastly, the proposed model could perform the reliable classification of all surfaces by means of temporal and spectral signal analysis. Ordered grouping between different surfaces with similar characteristics could also be observed. This gives consistency to the results and seems to advance a stable behavior for systems fed by the proposed model. Figure 10 shows that the cases of confusion in estimation were with surfaces that presented a profile similar to the desired one.

## 7. Conclusions

This paper proposes the use of the self-organizing map (SOM) for the identification of road surfaces in vehicles by acquiring the generated vibration in the suspension due to tyre rolling. Data analysis and decision management in control systems require the use of techniques that process information and generate sufficient knowledge to successfully execute a task. Nowadays, it is fundamental for technological development in the automotive industry to incorporate algorithms based on machine learning, deep learning, and similar techniques that help in this aspect. Making use of these techniques, analysis and extraction of signal characteristics are carried out for surface identification.

This procedure is useful to have continuous estimation of the road surface on which the vehicle is moving without involving the use of complex systems such as image analysis or acoustic techniques that may have interference with the environment. The algorithm acts on the unsupervised-learning paradigm by generating clusters with the different kinds of analyzed data. The power of this algorithm is based on agile analysis and the dimensional reduction of working data.

The proposed method was tested by conducting experiments on six different surfaces. Evaluation of the results obtained by the classification algorithm was carried out using the test data. The effectiveness of the proposed model was demonstrated by the achieved classification ratios (88.54%) and by the comparison with other classification algorithms.

In future work, several main lines of continuation will be considered. First, extending the working datasets with tests on a wider range of surfaces and at different speeds. The aim of this first step is to increase the knowledge base of the model and thus achieve more robust results. Second, in order to check the transversality of the created model, tests will be carried out with different vehicles or configuration parameters. Lastly, the real-time performance of the proposed classifier integrated in vehicle systems will be evaluated.

## Figures and Tables

**Table 1:** Description of different tested surfaces.

Surface	Description
Dry asphalt	Smooth dry asphalt with no major cracks or damage.
Wet asphalt	Smooth asphalt in good condition with water accumulation.
Poor asphalt	Dry asphalt with major cracks and holes.
Pavement	Smooth cement surface in good condition and without any damage.
Concrete	Uniform and clean concrete surface.
Gravel	Loose engraved surface and uneven distribution.

**Table 2:** Dimensional range of defined textures for a surface.

Texture	Wavelength (mm)
Megatexture	50–500
Macrotexture	0.5–50
Microtexture	0.0–0.5

**Table 3:** Specifications of the triple-axis accelerometer in MPU-9250.

Feature	Values
Operation voltage	3.3 V–5 V
Normal operating current	450 μA
Accelerometer Range	±2 g, ±4 g, ±8 g, ±16 g
Sensitivity Scale Factor	16,384 LSB/g, 8192 LSB/g, 4096 LSB/g, 2048 LSB/g
AD converter	16-bits (digital output)
Output data rate	1 kHz
Communication interface	I2C (Fast-mode 400 kHz)

**Table 4:** Summary of analyzed signal characteristics.

Time Domain			Frequency Domain		
**Param.**	**Feature**	**Definition**	**Param.**	**Feature**	**Definition**
$p_{t}\left(1\right)$	Standard deviation	$\sqrt{\frac{1}{n-1}\sum_{i=1}^{n}{\left|x_{i}-\mu \right|}^{2}}$	$p_{fa}\left(1\right)$	Mean	$\frac{1}{n}\sum_{i=1}^{n}\left(A_{i}\right)$
$p_{t}\left(2\right)$	Variance	$\frac{1}{n-1}\sum_{i=1}^{n}{\left|x_{i}-\mu \right|}^{2}$	$p_{fa}\left(2\right)$	Root mean square	$\sqrt{\frac{\sum_{i=1}^{n}\left(f_{i}^{2}A_{i}\right)}{\sum_{i=1}^{n}A_{i}}}$
$p_{t}\left(3\right)$	Root mean square	$\sqrt{\frac{1}{n}\sum_{i=1}^{n}{\left(x_{i}\right)}^{2}}$	$p_{fa}\left(3\right)$	Standard deviation	$\sqrt{\frac{\sum_{i=1}^{n}\left({\left(f_{i}-\mu_{f}\right)}^{2}A_{i}\right)}{\sum_{i=1}^{n}A_{i}}}$
$p_{t}\left(4\right)$	Square-root amplitude	${\left(\frac{1}{n}\sum_{i=1}^{n}\sqrt{|x_{i}|}\right)}^{2}$	$p_{fa}\left(4\right)$	Center	$\frac{\sum_{i=1}^{n}\left(f_{i}A_{i}\right)}{\sum_{i=1}^{n}A_{i}}$
$p_{t}\left(5\right)$	Maximum	$\max(|x_{i}|)$			
$p_{t}\left(6\right)$	Skewness	$\frac{\frac{1}{n}\sum_{i=1}^{n}{\left(x_{i}-\mu \right)}^{3}}{{\left(\sqrt{\frac{1}{n}\sum_{i=1}^{n}{\left(x_{i}-\mu \right)}^{2}}\right)}^{3}}$	$p_{fp}\left(1\right)$	Mean	$\frac{1}{n}\sum_{i=1}^{n}\left(P_{i}\right)$
$p_{t}\left(7\right)$	Kurtosis	$\frac{\frac{1}{n}\sum_{i=1}^{n}{\left(x_{i}-\mu \right)}^{4}}{{\left(\sqrt{\frac{1}{n}\sum_{i=1}^{n}{\left(x_{i}-\mu \right)}^{2}}\right)}^{2}}$	$p_{fp}\left(2\right)$	Maximum	$\max(P_{i})$
$p_{t}\left(8\right)$	Crest factor	$\frac{p_{t5}}{p_{t4}}$	$p_{fp}\left(3\right)$	Root mean square	$\sqrt{\frac{1}{n}\sum_{i=1}^{n}{\left(P_{i}\right)}^{2}}$
$p_{t}\left(9\right)$	Clearance factor	$\frac{p_{t5}}{p_{t3}}$	$p_{fp}\left(4\right)$	Square-root amplitude	${\left(\frac{1}{n}\sum_{i=1}^{n}\sqrt{|P_{i}|}\right)}^{2}$
$p_{t}\left(10\right)$	Shape factor	$\frac{p_{t4}}{\frac{1}{n}\sum_{n=1}^{n}\left|x_{i}\right|}$	$p_{fp}\left(5\right)$	Standard deviation	$\sqrt{\frac{1}{n-1}\sum_{i=1}^{n}{\left|P_{i}-\mu_{p}\right|}^{2}}$
$p_{t}\left(11\right)$	Impulse	$\frac{p_{t5}}{\frac{1}{n}\sum_{n=1}^{n}\left|x_{i}\right|}$			

**Table 5:** Range of SOM configuration parameters.

Parameters	Values
Inputs	1–20
Window (s)	0.1–0.3
Dimensions (n × n)	10–20
Initial neighbors	3–15
CoverSteps	200–600

**Table 6:** Comparative ratios with other classifiers.

Classifiers	Training Phase		Test Phase		
	Accuracy (%)	Execution Time (s)	Accuracy (%)	Execution Time (ms)	Execution Ratio
		(138 inputs)		(96 inputs)	
Proposed SOM	83.33	1.69	88.54	3.20	-
Tree fine	76.30	1.21	88.54	5.33	1.67
Tree coarse	66.13	0.90	75.00	6.15	1.92
Discriminant linear	87.92	0.83	91.67	7.51	2.35
Discriminant quadratic	81.10	0.76	92.71	10.22	3.19
Gaussian naïve Bayes	79.70	0.61	89.58	8.17	2.55
Kernel naïve Bayes	79.35	1.70	88.54	57.25	17.89
SVM linear	82.78	1.51	88.54	26.74	8.36
SVM quadratic	80.06	2.41	92.71	28.24	8.82
SVM cubic	78.84	1.43	91.67	32.65	10.20
SVM fine Gaussian	81.42	1.58	85.42	42.25	13.20
SVM coarse Gaussian	72.54	1.22	82.29	38.66	12.08
KNN fine	76.70	1.17	84.38	9.60	3.00
KNN coarse	16.95	2.02	40.63	17.48	5.46
KNN cosine	73.36	1.94	76.04	11.96	3.74
KNN cubic	76.74	1.88	87.50	16.84	5.26
KNN weighted	77.48	1.83	88.54	11.25	3.52
Bagged tress	82.40	3.03	90.63	46.83	14.63
Subspace discriminant	83.88	2.22	91.67	72.18	22.56
Subspace KNN	78.97	2.17	85.42	88.32	27.60
